# Association between telomere length and the risk of colorectal cancer: a meta-analysis of observational studies

**DOI:** 10.1186/s12885-016-2997-3

**Published:** 2017-01-05

**Authors:** Cho Naing, Kyan Aung, Pei Kuan Lai, Joon Wah Mak

**Affiliations:** 1 School of Postgraduate Studies, International Medical University (IMU), Kuala Lumpur, 57000 Malaysia; 2 School of Medicine, International Medical University (IMU), Kuala Lumpur, Malaysia

**Keywords:** Telomere, Colorectal cancer, Association, Meta-analysis

## Abstract

**Background:**

Human chromosomes are capped and stabilized by telomeres. Telomere length regulates a ‘cellular mitotic clock’ that defines the number of cell divisions and hence, cellular life span. This study aimed to synthesize the evidence on the association between peripheral blood leucocytes (PBL) telomere length and the risk of colorectal cancer (CRC).

**Methods:**

We searched relevant studies in electronic databases. When two or more observational studies reported the same outcome measures, we performed pooled analysis. All the analyses were performed on PBL using PCR. The odds ratio (OR) and its 95% confidence interval (CI) were used to assess the strength of association.

**Results:**

Seven studies (with 8 datasets) were included in this meta-analysis; 3 prospective studies, 3 retrospective studies and 1 study with a separate prospective and retrospective designs. The pooled analysis of 4 prospective studies (summary OR 1.01, 95% CI: 0.77–1.34, *I* ^2^:30%) and 4 retrospective studies (summary OR 1.65, 95% CI: 0.96–2.83, *I* ^2^:96%) showed no relationship between PBL telomere length and the CRC risk. A subgroup analysis of 2 prospective studies exclusively on females also showed no association between PBL telomere length and the CRC risk (summary OR, 1.17, 95% CI:0.72–1.91, *I* ^2^:57%).

**Conclusion:**

The current analysis is insufficient to provide evidence on the relationship between PBL telomere length and the risk of CRC. Findings suggest that there may be a complex relationship between PBL telomere length and the CRC risk or discrepancy between genetics, age of patients and clinical studies. Future well powered, large prospective studies on the relationship between telomere length and the risk of CRC, and the investigations of the biologic mechanisms are recommended.

**Electronic supplementary material:**

The online version of this article (doi:10.1186/s12885-016-2997-3) contains supplementary material, which is available to authorized users.

## Background

Human chromosomes are capped and stabilized by telomeres, which not only protect them from damage but also have a role in regulating cellular senescence. After reaching a critical length, telomeres experience a double DNA change and cells will eventually enter (replication) senescence or cell death [1, 2], which may be due to a loss of chromosomal integrity [3]. Laboratory observations showed that telomere of human somatic cells act as a “mitotic clock”, shortening with advancing age [1]. The exhaustion of proliferative potential of telomere, which is known as cellular senescence, occurred when telomeres cannot fulfil their normal protective functions [4].

There were an estimated 14.1 million cancer cases around the world in 2012. Of these, colorectal cancer (CRC) was the third most common cancer, accounting 1.36 million new case [5]. A considerable number of studies investigated the association between telomere length and human cancers including CRC. However, these individual studies reported inconsistently. Previous reviews in telomere sizes in cancer have reported CRC as a part [6–8], or were narrative reviews [9, 10]. Human telomere length vary with age or cell types [11] and animal models have shown that this may have diverse effects in various organ systems [12]. Since the publication of these reviews, there has been a surge of published studies which assessed the association between telomere length and CRC. Thus, a meta-analysis addressing CRC and telomere length would be a useful addition to the current information in this area. Meta-analysis is a particular statistical strategy for bringing together the results of several studies (i.e. independent but ‘comparable’ studies) to produce a single estimate [13]. On the whole, the objective of the present study was to synthesize the evidence of published studies on the association between peripheral blood leucocytes (PBL) telomere length and the CRC risk.

## Methods

The present study adhered to the preferred reporting items for systematic reviews and meta-analyses (PRISMA) statements [14].

### Study search

We searched the relevant studies in the electronic databases such as PubMed, EMBASE, CANCERLIT, DARE (Database of Abstracts of Reviews of Effects), CINAHL, Web of Knowledge and Google Scholar. In initial searches, we used the broad search strategy ‘telomere’ OR ‘telomere length’ AND ‘cancer’ OR ‘carcinoma’ AND ‘risk’ OR ‘epidemiology’ OR ‘pooled analysis’ OR ‘colon cancer’ OR ‘rectum cancer’ OR ‘colorectal cancer’. We modified the search strategy according to the requirements of different databases. Search was limited to publications in English through 7^th^ January 2016. We manually checked the reference lists of the relevant reviews and the included studies to find additional studies which could not be captured in electronic search.

### Study selection

Studies were eligible if they (i) reported on associations between baseline telomere length and CRC (as defined by histology), (ii) included controls (in case-control studies) who were cancer-free or healthy person, (iii) selected participants (in cohort studies) with pre-existing CRC, (iv) selected samples from PBL, and (v) provided sufficient data to estimate the strength of association.

As described elsewhere [15] both prospective and retrospective designs were considered. In retrospective studies, telomere length was measured in DNA samples collected after diagnosis of CRC. In prospectively designed studies, this was done from participants prior to diagnosis or development of CRC. Retrospective studies included case-control and cross-sectional studies, while prospective studies included nested case-control and prospective cohort studies. Studies were excluded if they did not meet the inclusion criteria. For studies that enrolled overlapping participants, only the studies with larger samples were included.

### Data extraction

Two authors independently screened the titles and abstracts, and assessed the full text, if deemed relevant for this review. Any discrepancy was resolved by discussion. The selected articles were reviewed to determine their eligibility for the current review. The data from the included studies were extracted using a piloted data extraction sheet. We extracted the following information from each of the included studies: author, year of publication, year of study, country, study design, type of CRC (i.e. colon cancer, rectal cancer, CRC), sample type, sample source, assay and outcomes reported. Any disagreement in data extraction was resolved by consensus or involving a senior author.

### Methodological assessments

One author appraised the methodological quality of each included studies using Newcastle-Ottawa Scale (NOS) [16]. This was cross-checked by another author. The study quality rating of each study was judged based on the participants’ selection (four stars), the comparability of the study groups (two stars) and the assessment of exposure (three stars). The highest total score for a study was nine. Any discrepancy between the two assessors was resolved by consensus and by taking advice from a senior author.

### Statistical analysis

We extracted study specific OR and corresponding 95% confidence interval (CI) from the included studies (i.e. for the association between shortest Q4 vs longest Q1, Q1 vs Q4 or below vs above the median length). We assumed that the relative risk (RR) from cohort studies approximates the odds ratio (OR) from case-control studies [17]. We transformed OR and its 95% CI into natural log OR and its standard error (SE) of log OR before meta-analysis [18]. Statistical heterogeneity between studies was assessed with the *I* ^2^ test, which indicates the variation in effect estimates due to (true) heterogeneity rather than within-study error [19]. A value of ≥50% indicates substantial heterogeneity [18, 19]. A random-effects model with inverse variance weighting was used to calculate the pooled OR and its 95% CI as there was substantial heterogeneity between studies [20]. Otherwise, we used a fixed-effect model.

All statistical analyses were performed with RevMan 5.3 [21]. Dataset is freely available from the corresponding author (CN) for non-commercial purpose.

## Results

### Study characteristics

The study selection process is provided in a flowchart (Additional file 1: Figure S1). After screening titles and abstracts, 20 full texts were retrieved; seven studies (with 8 data sets) [22–28] were selected for inclusion in this meta-analysis. Thirteen studies [29–41] were excluded as they did not meet the inclusion criteria. Reasons for the exclusion were summarized in Additional file 2: Table S1.

Table 1 presents the characteristics of the included studies. Three studies were prospectively designed studies [22, 23, 25], three studies were retrospective studies [26–28], and one study was done with prospective and retrospective designs, separately [24]. Two studies assessed exclusively with female participants [23, 25], while one study with males [22]. These studies were published between 2009 and 2014. Age of participants ranged from 18 to 82 years.

**Table 1:** Characteristics of the included studies

Author, publication yr, [Ref no]	Country	Site	Study year	Design	Pros/retro	Case selection	Control selection	Sample size_case/control	Matching	Male%	Mean age ± SD^b^	DNA source	Measurement technique	Adjusted factors^d^	Estimate of risks	Remarks
Zee, 2009 [22]	USA	78.8% colon; 21.2%:rectum	1982	nested	pros	Ca (PHS cohort)	healthy men	191/306	age ±2years, smk, follow-up length	all males	60.5 ± 8.66	PBL	qPCR	BMI, al, ex	0.8 (0.55–1.16)	50.3%: with aspirin use
Lee, 2010 [23]	USA	76.3% colon; 23.7%:rectum	Dec 2005	nested	pros	Ca (WHS cohort)	healthy women	134/357	age ±2years,smk, follow-up length	all females	60.1 ± 8.68	PBL	qPCR	age, smk, BMI, colorectal polyp, al, ex, pmp, HRT	0.94 (0.65–1.37)	35.1% with HRT
Pooley, 2010 [24]	UK	CRC/ADC	Jan 1998 -Dec 2003	nested	pros	Ca ^c^ (EPIC)	healthy	185/406	age, gender	female control	45–75	PBL	qPCR	study plate	1.13 (0.54–2.36)	
Pooley, 2010 [24]	UK	CRC/ADC	March 2001-Feb 2004	CC	retro	Ca registry (SEARCH)	healthy	2249/2161	age, gender	female control	18–69	PBL	qPCR	study plate	3.14 (1.77–2.58)	
Cui, 2012 [25]	China	CRC	Dec 1996- May 2000	nested	pros	Ca (SWHS)	healthy	441/549	age, date, time ^a^, meal, antibiotic, mp	all females	58.5 ± 8.7	PBL	qPCR	age, date, time	1.56 (0.92–2.64)	part of 74942 cohort
Pellat, 2013 [26]	USA (Utah)	CRC	1991-94	CC (DALS)	retro	Ca (DALS)	driver license & social security lists	249/374 (1991-94) (colon) 276/372 (1997–2002) (rectal)	age, sex	54%	30–79	Cell line (colon), PBL (rectal)	qPCR	age, sex, BMI, smk, aspirin/NSAID, trans-fatty acid intake	0.96 (0.82–1.0)	44% on HRT, h/o aspirin, smk
Boardman, 2014 [27]	USA	colon polyps & CRC	2000+	CC	retro	Ca registry & Ca (Mayo biobank)	healthy ^f^	598/2212	age, gender, location.	47% (colon); 58% (Mayo)	48 (19–69)	PBL	qPCR	multiple	3.53 (1.35–9.24)	chemoradio Rx; naive Ca
Qin, 2014 [28]	China	CRC	Jan 2007- Dec 2010	CC	retro	Ca, hospitalised	unrelated healthy ^e^	628/1256	age ± 5 years, gender	54.1%	58,8 ± 11.8	PBL	qPCR	smk, Al, age, sex.	1.47 (1.09–1.99)	chemo-radio Rx & radio Rx; naive Ca

^a^ matched with date and time of sample collection; ^b^: mean age of cases: ^c^: cancer free at the baseline assessments, but diagnosed as CRC at least 6 months after; ****: overall from 3 sources; *****: CRC & without CRC; *Al* alcohol use, *BMI* body mass index, *Ca* cancer, *CRC* colorectal cancer, *CC* case-control study design, *healthy* healthy controls, *h/o* history of; nested: nested case-control study, *HRT* hormone therapy, *mp* menopause, *mth* months; nested: nested case-control, *PHS* prospective physicians’ health study, *qPCR* quantitative PCR, *EPIC* European prospective investigation into cancer, *PBL* peripheral blood leucocyte, *pmp* post menopause, *Pop* population, *Ref* Reference, *smk* smoking status, *SWHS* Shanghai women’s health study, ^d^: further controlling factors to case-control status,^e^: based on authors address; ^f^: unrelated residents; *NA* not available or not relevant, as appropriate, *Rx* treatment or therapy as appropriate

Two studies each were done in China [25, 28] and the UK [24, 25] The remaining three studies were from the United States [22, 23, 27]. Three studies [22, 23, 27] have provided separate components in CRC, indicating most were colon cancer (70.3–78.8%) and the rest were rectal cancer (21.2–29.7%). In all studies, DNA sources was extracted from PBL and used qPCR to measure the telomere length. The Pellatt study used immortalized cell lines for colon cancer and PBL for rectal cancer [26]. Methodological quality of the included studies was provided (Additional file 3: Table S2). All studies were high in methodological quality, achieving 7–9 stars [22–28].

### Summary estimates

**Fig. 1:**
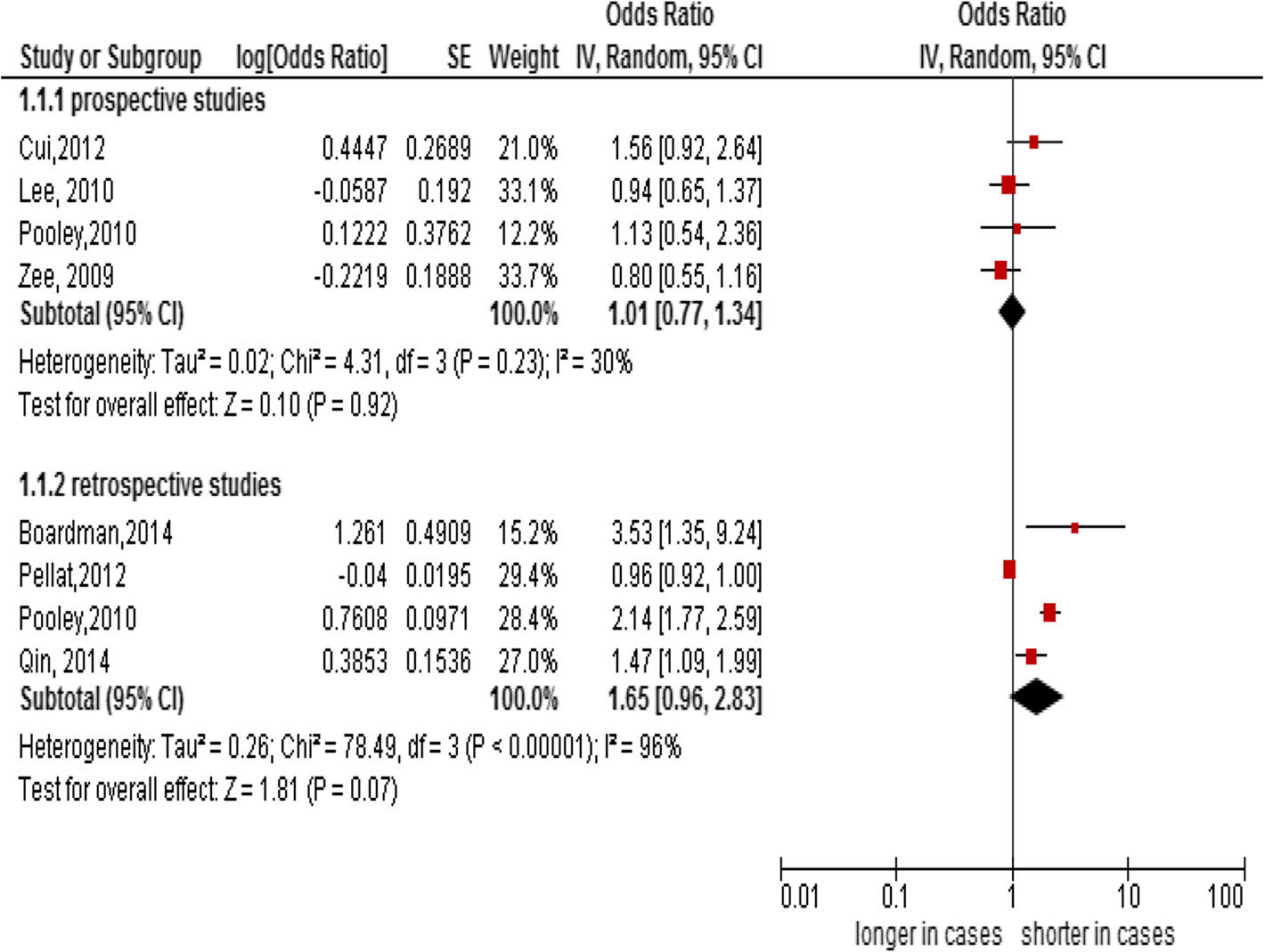
Forest plot of association between telomere length and the colorectal cancer risk

Overall, there is no significant relationship between telomere length measured in PBL DNA and the CRC risk in both retrospective and prospective studies. The summary OR in 4 retrospective studies was 1.65 (95% CI: 0.96–2.83) with substantial statistical heterogeneity (*I* ^2^:96%). The summary OR (1.01, 95% CI: 0.77–1.34) in four prospective studies was also with significant heterogeneity (*I* ^2^:30%) (Fig. 1).

### Subgroup analysis

**Fig. 2:**
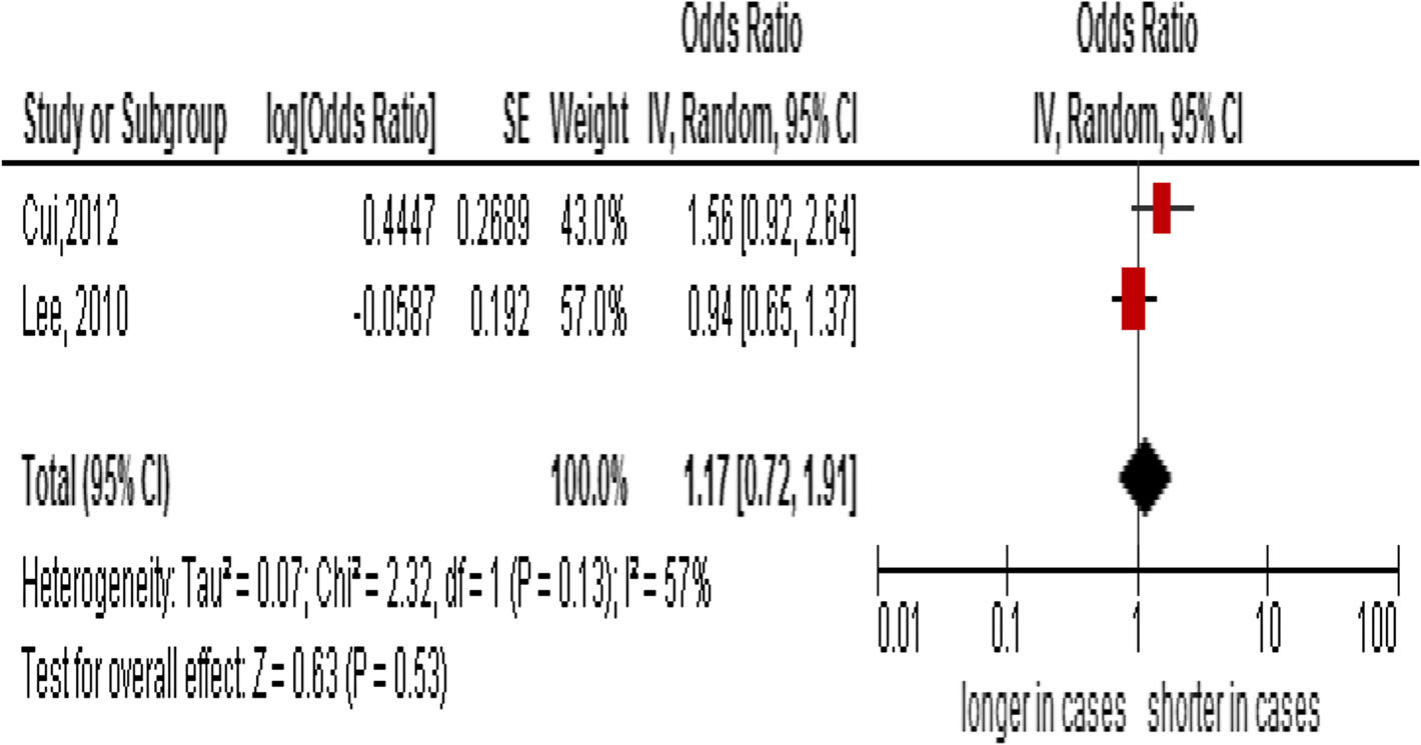
Forest plot of a subgroup analysis solely on female participants

When we analyzed two prospective studies solely on female participants [23, 25], there also was no significant association between telomere length and the CRC risk (summary OR, 1.17; 95% CI:0.72–1.91, *I* ^2^:57%) (Fig. 2). Due to a paucity of data, we were not able to perform subgroup analysis with age groups.

## Discussion

Based on the available data, the present study has provided insights into the relationship between PBL telomere length and the CRC risk. Due to small number of studies the results from the current analysis is insufficient to provide evidence on the role of PBL telomere length and CRC risk.

### Telomere length in CRC

Findings in this review showed there is no significant relationship between telomere length measured in PBL DNA and the CRC risk in both retrospective and prospective studies. There are many possible reasons for such relationship. The risk of CRC might be elevated by shorter or longer length of telomere, indicating a U-shape association [25]. It is possible that loss of telomeric DNA in relation to degradation or incomplete replication is apparently balanced by telomere elongation [1, 42, 43]. This also implied that telomere length in an ‘appropriate range’ may be necessary to maintain chromosomal stability and normal programmed cell death - functions which are protective against tumour development [25, 44]. It has been hypothesized that cells with long telomeres may favour a delayed cell senescence and apoptosis, leading to an increased chance of various genetic and environmental insults and subsequent accumulations of genetic abnormalities attributed to a higher risk of carcinogenic transformation [25].

On the other hand, the relationship between short telomere length and the CRC risk is biologically plausible [28]. A large proportion of human cancers are made up of cells with very short telomeres (5 kb), which is attributable to telomere dysfunction [45]. Although the exact mechanism is not fully understood, it is possible that, in some situations, cells with critically short telomere length may reactivate the telomerase enzyme, and this further promotes malignant transformation [28]. The putative pathway for telomere-associated neoplasia is that shortening of telomeres in the colorectal mucosa increases the chromosomal susceptibility to instability [40] as well as the microsatellite instability [47].

Reasons for no significant associations in our findings also rest on discrepancy of the study participants such as gender, age, life styles and the study design related factors (e.g. sample size, prospective or retrospective study). For instance, an association of telomere length and the CRC development in age-depending manner was shown in an individual study included in this analysis [28]. Older patients with shorter telomeres may be prone to an increased risk for various types of cancers due to telomere crisis [28] and subsequent chromosomal aberrations [25] and the diseases that result [25]. Extremely long telomere in younger individuals may be indicative of dysregulation in telomere regulation process [28].

We found a change in the effect estimates in a subgroup analysis solely with females. This highlighted that hormonal influence (estrogen) on the length of telomere needed to be given due attention in interpreting the results. Studies documented that the rate of terminal restriction fragment length shortening per year in men was significantly greater than that in women [45]. An estrogen-responsive element is present in telomerase reverse transcriptase (hTERT), so the hormone can stimulate telomerase [46]. This might have reduced the shortening of telomere. Another possible mechanism is in the light of reactive oxygen species (ROS), which are number of reactive molecules and free radicals derived from molecular oxygen. As such, hydrogen peroxide and other ROS accelerate telomere erosion at least in cultured cells. Women are known to produce fewer reactive oxygen species [42] and this might contribute to less telomere attrition.

Most of the primary studies in this review have estimated an individual’s telomere length from a single blood collection. In a study, multiple measurements showed a good reliability (intraclass correlation coefficient 0.64) indicating a one-time measurement is a good representation of an individual’s telomere length within a short period of time [48]. Hence, a one-time determination in the primary studies would not affect the confidence in the effect estimate of the current review.

### Study limitations and strengths

There are some limitations that need to be acknowledged. Although we had done an exhaustive literature search, it was possible that some publications might have been overlooked, especially when abstracts in English were not available. Variation in sample collection time in relation to study design among included studies is a concern. Samples in the prospective study were collected (many) years before diagnosis and therefore changes in participant’s diet and lifestyle related factors and drug history (e.g. treatment with estrogen in women) overtime could have influenced telomere length, or conversely telomere length could be influenced by diagnosis and treatment in the retrospectively designed study [24, 27].

Variation in measurement methods of telomere lengths in the primary studies could have affected the effect estimates. This was, however, not the case in the present review as all studies applied qPCR for the measurement of telomere length. Stage-specific relationship could not be analysed as staging of disease with Dukes’ or TNM systems had been provided inconsistently in a few studies. This might lead to a misclassification bias in interpreting the results.

Since relevant data were limited, we could not adjust for confounding factors. For instance, age of patients was likely to be a confounding factor for the association between PBL telomere length and the CRC risk. It is difficult to render the pooled analysis stratified by age groups, which were not consistently classified among these studies. Individual studies [45, 49] as well as a systematic review had suggested an interaction between aspirin or non-steroidal anti-inflammatory drug use [49, 50] and genes encoding transcription factor [49] or a combined effect of life style with gene encoding [27] in oncogenesis, inflammatory and drug metabolic pathways in relation to risk of CRC [50]. Hence, our results might be influenced by these confounding factors.

Due to a small number of studies with limited sample sizes, there is a limited power to detect a true difference of telomere length between cases and controls. It calls for future well designed, large prospective studies in this field.

## Conclusion

The current analysis is insufficient to provide evidence on the relationship between PBL telomere length and the risk of CRC. Findings suggest that there may be a complex relationship between PBL telomere length and CRC risk, suggesting a U-shaped relationship or discrepancy between genetics, age of patients and clinical studies. Future well powered, large prospective studies on the relationship between PBL telomere length and the risk of CRC, and the investigations of the biologic mechanisms are recommended.

## Additional files

Additional file 1: Figure S1. Study selection process. (DOC 29 kb)
Additional file 2: Table S1. Excluded studies and reason for exclusion. (DOC 26 kb)
Additional file 3: Table S2. The methodological quality of the included studies. (DOC 41 kb)

## Abbreviations

CI: Confidence interval
CRC: Colorectal cancer
OR: Odds ratio
PBL: Peripheral blood leucocytes
ROS: Reactive oxygen species
SE: Standard error
