# Supplementary material for: Association between telomere length and the risk of colorectal cancer: a meta-analysis of observational studies
Source: BMC Cancer. 2017 Jan 5;17:24. doi: 10.1186/s12885-016-2997-3 (PMC5216529; doi:10.1186/s12885-016-2997-3)
Supplement: Additional file 1: Figure S1. — Study selection process. (DOC 29 kb) [file 12885_2016_2997_MOESM1_ESM.doc]

Additional Figure 1 Study selection process

**Screening**

**Included**

**Eligibility**

**Identification**

Records identified through database searching
(n = 452)

Additional records identified through other sources
(n = 1 )

Records after duplicates removed
(n = 376)

Records screened
(n =137 )

Records excluded
(n = 239 )

Full-text articles assessed for eligibility
(n = 20 )

Full-text articles excluded, with reasons
(n = 13 )

Studies included in quantitative synthesis (meta-analysis)
(n = 7 )
