# Supplementary material for: Association between telomere length and the risk of colorectal cancer: a meta-analysis of observational studies
Source: BMC Cancer. 2017 Jan 5;17:24. doi: 10.1186/s12885-016-2997-3 (PMC5216529; doi:10.1186/s12885-016-2997-3)
Supplement: Additional file 2: Table S1. — Excluded studies and reason for exclusion. (DOC 26 kb) [file 12885_2016_2997_MOESM2_ESM.doc]

Table S1 Excluded studies and reason for exclusion

| Study | Ref. | Reason* |
| --- | --- | --- |
| Engelhardt et al, 1997 | 29 | assessed on CRC progression |
| Engelhardt et al, 1997 | 30 | assessed on CRC progression |
| Takagi et al, 1999 | 31 | no controls from PBL |
| Takagi et al, 2000 | 32 | assessed on microsatellite instability |
| Gertler et al, 2002 | 33 | assessed on CRC prognosis |
| Gertler et al, 2004 | 34 | assessed on CRC prognosis |
| Garcia-Aranda et al, 2006 | 35 | not a human study |
| O'Sullivan et al, 2006 | 36 | Not a human study |
| Bautista et al, 2009 | 37 | assessed on adenomatous polyps |
| Jones et al,2012 | 38 | assessed on polymorphisms |
| Riegert-Johnson et al,2012 | 39 | assessed on advanced adenomas |
| Chen et al,2014 | 40 | assessed on CRC prognosis |
| Segu et al, 2014 | 41 | no data on association |

*not meeting inclusion criteria and/or meeting exclusion criteria
