# Supplementary material for: Association between telomere length and the risk of colorectal cancer: a meta-analysis of observational studies
Source: BMC Cancer. 2017 Jan 5;17:24. doi: 10.1186/s12885-016-2997-3 (PMC5216529; doi:10.1186/s12885-016-2997-3)
Supplement: Additional file 3: Table S2. — The methodological quality of the included studies. (DOC 41 kb) [file 12885_2016_2997_MOESM3_ESM.doc]

Table S2 The methodological quality of the included studies

| Author, year [ref no.] | selection | | | | comparability | | outcome | | | total stars |
| --- | --- | --- | --- | --- | --- | --- | --- | --- | --- | --- |
|  | representativeness | selection of non-exposed cohort | ascertainment of exposure | outcome of interest is not at the start of study | most important factor | additional factor | assessment of outcome | was follow-up long enough for outcomes to occur | adequacy of follow up of cohorts |  |
| Zee, 2009 [22] ^■^ | * | * | * | * | * |  | * | * | * | 8 |
| Lee, 2010 [23]^■^ | * | * | * | * | * |  | * | * | * | 8 |
| Pooley,2010 [24] | * | * | * | * | * |  | * | * | * | 8 |
| Cui,2012 [25] | * | * | * | * | * | * | * | * | * | 9 |
| Pellatt,2012  [26] | * | * | * |  | * |  | * | * | * | 7 |
| Boardman, 2014 CC [27] | * | * | * |  | * |  | * | * | * | 7 |
| Qin, 2014  [28] ^■^ | * | * | * |  | * |  | * | * | * | 7 |

^■^Only one particular ethnic group.
